# Socio-economic inequalities in curative health-seeking for children in Egypt: analysis of the 2008 Demographic and Health Survey

**DOI:** 10.1186/s12913-015-1150-3

**Published:** 2015-10-24

**Authors:** Lenka Benova, Oona M. R. Campbell, George B. Ploubidis

**Affiliations:** Department of Infectious Disease Epidemiology, Faculty of Epidemiology and Population Health, London School of Hygiene and Tropical Medicine, Keppel Street, London, WC1E 7HT United Kingdom; Department of Population Health, Faculty of Epidemiology and Population Health, London School of Hygiene and Tropical Medicine, Keppel Street, London, WC1E 7HT United Kingdom; Centre for Longitudinal Studies and Population Health and Statistics at the Department of Quantitative Social Science, UCL Institute of Education, London, WC1H 0AL United Kingdom

**Keywords:** Child health, Egypt, Socio-economic inequalities, Diarrhoea, Acute respiratory infection, Mediation analysis, Health-seeking behaviour, Care utilisation

## Abstract

**Background:**

The existence and magnitude of socio-economic inequalities in health-seeking behaviours for child curative care in Egypt and mechanisms underlying these associations have not been comprehensively assessed. This study examined whether socio-economic position (SEP) was associated with health-seeking behaviours for diarrhoea and acute respiratory infection (ARI) in children and explored potential mechanisms underlying these associations using mediation analysis.

**Methods:**

Children aged under-five years living with their mothers sampled by the 2008 Egypt Demographic and Health Survey were used to estimate the prevalence of diarrhoea and ARI in the two-week period preceding the survey. If either illness was reported, three dimensions of health-seeking were examined in adjusted mediation models, separately by illness: whether medical care was sought, whether such care was timely (within one day of symptom onset), and whether it was sought from private providers. Latent variables of parental socio-cultural capital and household-level economic capital were the main exposures of interest.

**Results:**

In the sample of 10,006 children, 8.4 % had diarrhoea and 7.6 % had ARI. Care was sought for 62.0 % of children with diarrhoea and 78.5 % with ARI; two-thirds of care-seeking for both illnesses was timely. More than 7 in 10 children who sought care were taken to private providers. Socio-cultural capital or economic capital were not independently associated with seeking care for either illness. Socio-cultural capital was positively associated with timely care-seeking, and economic capital was positively associated with private provider use in adjusted analyses for both illnesses.

**Conclusions:**

SEP was not a strong determinant of care-seeking for diarrhoea or ARI, but there was a modest positive effect of SEP on timely receipt of care and private provider use. Further research is needed to explore perceptions of illness severity and the availability and quality of care from public and private providers.

## Background

Coverage and equity of child health interventions have gained prominence in light of the aim of Millennium Development Goal 4 to reduce under-five mortality rate by two-thirds between 1990 and 2015. Egypt achieved large declines in infant and child mortality; infant mortality decreased from 62 to 25 per 1,000 live births between 1990 and 2006 [1]. During the same period, under-five mortality risk declined from 85 to 28 per 1,000 live births [1]. The decline in under-five mortality has been attributed to a host of health system interventions, including promotion of oral rehydration therapy through the National Control of Diarrhoeal Diseases Program in the 1980s, scale up of the Integrated Management of Childhood Illness protocol starting in 1999, increase in the proportion of children who received basic immunisations to near-universal levels, and coverage of children under-five in the Health Insurance Organisation health insurance scheme [2–7]. Additionally, social and infrastructural improvements in the education level of women, decrease in unwanted pregnancies, and access to improved water and sanitation have aided in reducing child mortality and morbidity.

Despite decreasing at an average annualized rate of −5.4 % between 2000 and 2013, an estimated 41,300 deaths under-five years occurred in Egypt in 2013 [8]. Mortality attributed to diarrhoea and pneumonia was estimated to account for 18 % of deaths among children under-five years in Egypt in 2010 [9]. However, a study employing verbal autopsy methods showed that diarrhoeal diseases and acute respiratory infection were the leading causes of mortality among children 1–4 years of age, accounting for more than 80 % of these deaths [10]. A better understanding of the determinants of health-seeking behaviours for curative care after the onset of these illnesses is crucial to developing strategies to further reduce under-five mortality.

However, population-level statistics fail to capture large geographic and socio-economic inequalities in the survival of children under-five, and slower rates of improvement in children from lower socio-economic backgrounds. The 2008 Egypt Demographic and Health Survey (DHS) estimated that in the 10 years preceding the survey, under-five mortality was 19 per 1,000 live births in the wealthiest quintile of households and 49 per 1,000 in the poorest quintile, and a geographic comparison showed that urban Lower Egypt had the lowest (18/1,000) and rural Upper Egypt the highest rate (46/1,000) [1].

Effective prevention and treatment of childhood illnesses, such as diarrhoea and pneumonia, can lead to further reductions in morbidity and mortality among children under-five years. However, their uptake and coverage rely on households’ perception of need, health-seeking behaviours, and on the quality of care received once a health provider is reached. On the 2008 DHS, 7.6 % of children under-five were reported to have had ARI and 8.4 % diarrhoea in the two-week recall period. The data showed no obvious differences in the period prevalence of these two illnesses between children from households in the various wealth quintiles [11]. This lack of socio-economic inequality in the crude measures of illness reporting potentially points to health-seeking behaviour and quality of care as important determinants of child health outcomes and survival in Egypt.

Socio-economic resources are well-established determinants of child care utilisation in low- and middle-income countries [12–14]. In Egypt, large gaps exist in the understanding of the extent of socio-economic inequalities in curative health-seeking behaviours for children. A 2014 systematic review of literature did not identify any published papers assessing health-seeking behaviours related to child illnesses using the 2008 Egypt DHS, or any studies that used adjusted analytical methods to examine the association between socio-economic position (SEP) and health-seeking behaviour for curative care in children on a nationally-representative sample in Egypt [15]. The only analysis of nationally-representative data of curative health-seeking for child illnesses used data from the 1995 and 2000 DHS rounds to estimate crude socio-economic inequalities based on the DHS wealth index [16]. This report by Gwatkin and colleagues found that the extent of socio-economic inequalities in seeking care decreased between the two surveys for acute respiratory infection (ARI), but increased for diarrhoea. They also showed that the inequality in choosing a private provider increased for both illnesses.

### Objectives

This study used the 2008 Egypt DHS to address three objectives. First, it described the prevalence of diarrhoea and ARI reported for children under-five years of age living in Egypt. Second, it characterised the process of health-seeking for children with these illnesses using three dimensions: whether medical care was sought, and if so, whether such care was timely (within one day of illness onset), and whether it was sought from public or from private providers. Third, it examined the association of SEP with progression through these dimensions of health-seeking. This objective was accomplished by using latent variables capturing two constructs of SEP in a mediation analysis which allowed an assessment of their relative importance as drivers of any inequalities identified.

## Methods

### Study sample

The analysis was based on the 2008 Egypt DHS, a nationally-representative survey of households that collected indicators of child health for all children under-five born to all ever-married women aged 15–49 living in sampled households. Children were included in the analysis if their mother was alive and resided in the sampled household and if the child was alive and co-resided with mother. If more than one child per household and per mother existed, all were included.

### Measures of SEP

The construction of the two continuous latent variables capturing socio-cultural capital and economic capital was described previously [17, 18]. Briefly, socio-cultural capital is thought to reflect knowledge, ability to access information, cognitive skills, exposure to authority, and ability to interact with institutions [19–22]. This latent variable was based on mother’s and father’s education, mother’s literacy, father’s occupational category and maternal working status. Economic capital captures the household-level material resources available to meet the direct and indirect costs of care [23]. It was constructed from ten binary variables, including utilities (water piped into dwelling, flush toilet), household ownership of assets (fridge, car, mobile, colour TV, water heater, automatic washing machine), ownership of a bank account, and existence of crowding in the residence. High latent variable scores represented higher SEP. Economic capital was considered a mediator of the association between socio-cultural capital and the health-seeking behaviour outcomes. Children were assigned the socio-cultural capital score of their parents and the economic capital score of their households.

### Health-seeking behaviour outcomes

**Fig. 1:**
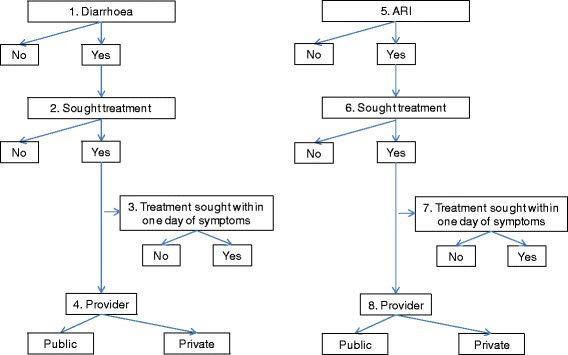
Dimensions of curative health-seeking behaviours for illnesses reported for children under age of 5 years in the two-week period preceding the survey

The first step in assessing health-seeking behaviours for curative care was to analyse the reporting of diarrhoea and ARI in the two-week period preceding the survey among children under-five years (0–59 months old). For each child in the sample, the mother was asked whether the child had diarrhoea in the two-week period preceding the survey. If the answer was affirmative, the questionnaire elicited whether there was blood in the child’s stool and whether advice was sought from any source. If the mother confirmed that help was sought, she was asked how many days elapsed between the onset of illness and the seeking of care, and listed all places that were approached for care (Fig. 1). When the sequence of questions about diarrhoea was completed, the mother was asked whether, in the same two-week period, the child had fever and whether the child had an illness with cough. If cough was reported, the mother was asked whether the child experienced rapid breathing or difficulty breathing, and if so, whether this fast or difficult breathing was chest- or nose-related, or both. If either fever or cough was reported, the sequence of three questions related to care-seeking (as detailed above for diarrhoea) was repeated (Fig. 1).

The analysis sample of health-seeking behaviours included children for whom diarrhoea or ARI were reported. ARI, a proxy for pneumonia, was defined as cough accompanied by short or rapid breathing which was chest-related. The severity of diarrhoea was assessed by the mother’s report of blood in the stool, a proxy for dysentery. For both diarrhoea and ARI, the report of fever during the two-week recall period was considered as an additional symptom, although from the design of the questionnaire it was not possible to ascertain whether, in cases where two or more symptoms were reported, such symptoms occurred at the same time. Binary variables capturing whether diarrhoea and ARI were reported or not were created, and separately for each reported illness, the health-seeking was characterised by whether or not help was sought. If help was sought, timely treatment was defined as seeking help within one day of symptom onset whether provider was public or private.

#### Provider type

The respondents were asked to recall all places where care was sought from the 15 detailed response options listed on the questionnaire. To describe the specific provider types approached for each illness, the responses were grouped into the following eight categories: government hospital (urban or rural), government health unit (urban or rural), other public (MCH center/health office/other government), providers from both sectors, private hospital/clinic, private doctor, private pharmacy, and other/NGO providers. The final binary (public or private) location of care outcome was private if one or more private providers, or a combination of public and private providers, were approached; and otherwise public. This categorisation mirrors methods employed previously in analysing multiple providers for antenatal care [17].

### Confounders

*A priori* confounders of the association between socio-cultural capital, economic capital and child health-seeking behaviours were identified from the existing literature [1, 2]. These included child sex and age group (in one-year intervals), mother’s age group (five-year intervals), preceding birth interval, and the mother’s status as the female head of household (yes/no). A variable capturing the number of children under-five years residing in the household derived from the household members roster (not necessarily children of the same mother) was included because a previous study from Egypt showed that the presence of another household member with diarrhoea was the strongest independent risk factor for diarrhoea [24]. The presence of several small children could potentially delay initial recognition of illness and the number of young children may influence the ability of the main caretaker to seeking care outside the household. Elements of availability of health services were captured in the residence variable (urban or rural).

A categorical variable capturing the month in which the interview was conducted (March, April or May/June) was considered a confounder in analysis of illness reporting due to the seasonal nature of childhood illness incidence [25, 26]. This variable was also included in analyses of seeking care from private provider due to the possible influence of seasonality on perception of illness severity [27]. In analyses of health-seeking for diarrhoea, the presence of blood in stool was included as a proxy for illness severity. Binary variables capturing whether the child suffered from ARI or fever during the two-week recall period were also included [28]. Likewise, in analyses of health-seeking for ARI, binary variables capturing whether the child had fever or diarrhoea during the recall period were included in multivariable analyses. Lastly, the multivariable logistic regression models for the outcome capturing whether treatment was sought at a private provider, timely seeking of treatment (within a day of illness onset) was included as a covariate.

### Statistical analysis

Descriptive, multivariable and mediation analyses were conducted separately for the three binary health-seeking behaviour outcomes available for each illness in Stata SE13. If a child was reported to have suffered from both diarrhoea and ARI during the recall period, he/she is included in both analysis samples. We accounted for the complex survey sampling design (clustering, stratification and weights) by using the *svyset* command. The proportion of missing data in the majority of the outcome variables was minimal and we utilised complete case analysis. The distribution of categorical and binary variables was described by percentages and compared using the Chi-square test. Continuous SEP variables were summarised using means and standard errors and differences tested using the *t*-test.

**Fig. 2:**
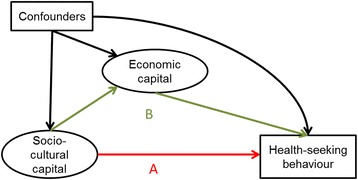
Conceptual path diagram of the structural model

The direct effects of both measures of SEP were modelled in logistic regression; odds ratio was the main effect estimate. Figure 2 shows the conceptual framework of the analysis in which socio-cultural capital can be directly or indirectly (through economic capital) associated with the outcome under examination. Continuous latent scores for both variables were entered in this adjusted mediation model, in order to jointly estimate their associations. The total effect of socio-cultural capital (sum of its direct and indirect effects) on binary outcomes was expressed as the sum of changes in the probability of outcome (*ΣΔp)* associated with a one unit increase in the standardised latent variable. The Stata *mediation* package was used [29].

### Ethics

The collection of the DHS data was approved by local authorities in Egypt; respondents’ informed consent was sought and data were anonymised. This secondary analysis of the data was approved by the Research Ethics Committee of the London School of Hygiene and Tropical Medicine, UK.

## Results

The 10,006 children included in the sample lived in 7,495 unique households; there were between one and four children under-five years living in these households. Descriptive characteristics of the three groups of children in the sample (all children, children with diarrhoea and children with ARI) are shown in Table 1. Children with either illness, and particularly children with diarrhoea, had a younger age distribution compared to all children in the sample. However, the gender composition of children with diarrhoea and of children with ARI did not differ significantly from all children. Mothers of children with diarrhoea were significantly younger than mothers of all children in the sample. Children with ARI were marginally more likely than all children to reside in urban areas. There was a strong crude association between the month of interview and likelihood of having diarrhoea and ARI; children with either illness were more likely to have been interviewed earlier in the year. The mean socio-cultural capital score in households of children with diarrhoea and ARI did not differ from mean scores of all children. The mean economic capital scores did not differ between children with diarrhoea and all children in the sample, but households of children with ARI had marginally lower mean economic capital scores than households among children.

**Table 1:** Distribution of demographic and socio-economic variables in samples

Characteristics		All children <5 years old	Children with diarrhoea	Children with ARI
	Sample size (n)	10,006	840	761
Child age (months)	0–5 (%)	10.5	15.1	11.3
	6–11	12.3	27.8	19.3
	12–23	21.0	27.9	26.7
	24–35	19.4	15.3	15.8
	36–47	18.8	7.5	13.6
	48–59	18.0	6.4	13.3
	*X* ^*2*^ p value*		*<0.001*	*<0.001*
Child sex	Male (%)	50.6	52.5	52.7
	Female	49.4	47.5	47.3
	*X* ^*2*^ p value*		*0.260*	*0.245*
Preceding birth interval	<24 months (%)	12.1	11.7	11.7
	24–47 months	33.5	32.1	36.4
	≥48 months	21.9	21.3	24.1
	Only child	32.5	34.9	27.8
	*X* ^*2*^ p value*		*0.527*	*0.039*
Mother’s age group	14–19 (%)	8.0	12.8	9.0
	20–24	33.1	34.4	30.8
	25–29	32.0	29.0	33.2
	30–34	16.6	16.0	17.6
	35–39	8.1	6.1	7.3
	40–49	2.2	1.7	2.1
	*X* ^*2*^ p value*		*<0.001*	*0.670*
Mothers’ household status	Female head (%)	81.9	81.2	81.8
	*X* ^*2*^ p value*		*0.626*	*0.947*
Number of children <5 in household	1 (%)	39.5	43.0	39.7
	2	44.2	41.4	43.7
	3 or 4	16.3	15.6	16.6
	*X* ^*2*^ p value*		*0.167*	*0.967*
Region	Urban (%)	36.5	39.5	41.3
	Rural	63.5	60.5	58.7
	*X* ^*2*^ p value*		*0.139*	*0.048*
Month of interview	March (%)	22.5	33.2	42.0
	April	51.9	44.8	44.1
	May/June	25.6	22.0	13.9
	*X* ^*2*^ p value*		*<0.001*	*<0.001*
Socio-cultural capital	Mean	0.016	−0.004	−0.044
	SE	0.013	0.026	0.030
	T test p value**		*0.502*	*0.150*
Economic capital	Mean	0.047	0.029	0.019
	SE	0.014	0.026	0.028
	T test p value**		*0.220*	*0.052*

*SE* standard error. Complex survey design (weighting, clustering and stratification) was accounted for in calculations of proportions and sample sizes reported. *ARI* cough with difficulty breathing which is chest related

*Testing the hypothesis that users children for whom symptoms of diarrhoea/ARI in the two-week period before the survey were reported were drawn from the same population as children without these symptoms

** *T* test p value testing that the difference in mean scores between children with and without symptoms of diarrhoea or ARI was 0

### Burden of illness

**Fig. 3:**
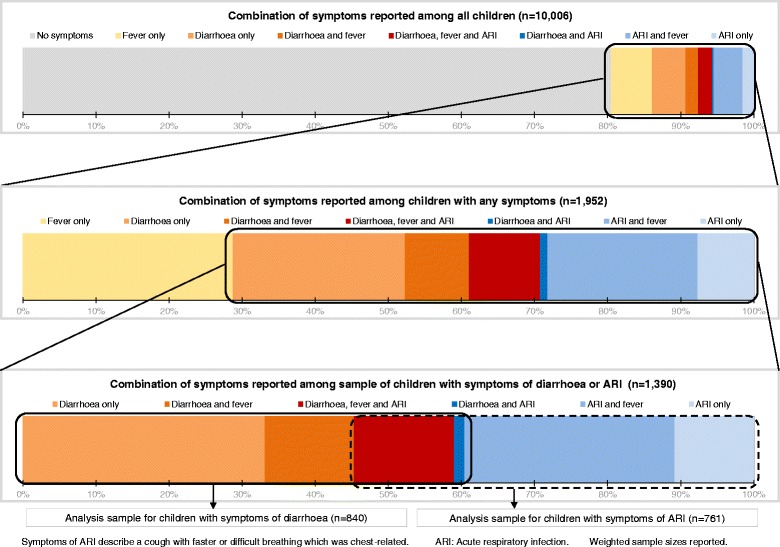
Samples of children according to combination of symptoms reported

Among all children, 8.4 % (95 % confidence interval [CI]: 7.8–9.1) were reported to have had diarrhoea and 7.6 % (95%CI: 6.9–8.4) to have had ARI. Figure 3 shows that 19.5 % of all children were reported to have experienced one or more of the three illnesses (fever, ARI, or diarrhoea) in the two-week recall period. Among the 1,952 children for whom one or more illness was reported, 28.8 % had only fever. Among the sample of 1,390 children with diarrhoea or ARI, half (56.1 %) had two or three illnesses. Among the 840 children for whom diarrhoea was reported, 42.9 % also had fever during the recall period. On the other hand, 77.6 % of the 761 children with ARI were reported to have had fever during the recall period.

### Health-seeking for ill children

Table 2 shows the samples of children analysed in the various dimensions of health-seeking, including definition of denominators, extent of missing data, and levels of health-seeking outcomes. Among children who had diarrhoea, care was sought for 62.0 % (95 % CI: 58.3–65.6). In the group of children with diarrhoea for whom care was sought, 61.8 % (95 % CI: 56.6–66.8) received timely treatment and 71.9 % (95 % CI: 67.2–76.2) were taken to a private sector provider. Among children with ARI, care was sought for 78.5 % (95 % CI: 74.9–81.8). Among children with ARI for whom treatment was sought, 62.0 % (95 % CI: 57.0–66.7) received timely treatment and 72.6 % (95 % CI:68.2–76.6) were taken to a private provider. Only a small proportion of children for whom care was sought were taken to more than one provider (4.3 % for diarrhoea and 2.9 % for ARI), and declined further when the proportion seeking care from both sectors was assessed (2.5 % for diarrhoea and 1.8 % for ARI).

**Table 2:** Analysis samples and child health-seeking behaviours among children <5 years of age

Health-seeking behaviour outcome		Samples and missing data^a^				Distribution of outcome in analysed sample and 95 % CI
		Eligible sample	Eligible sample size	Missing data (%)	Analysed sample size	
*Diarrhoea*						
1.	Reported illness	All children	10,006	0.12 %	9,994	8.4 % (7.8–9.1)
2.	Sought treatment	All children for whom symptoms of diarrhoea were reported	840	-	840	62.0 % (58.3–65.6)
3.	Timely treatment	All children for whom symptoms of diarrhoea were reported and who sought treatment	521	0.38 %	519	61.8 % (56.6–66.8)
4.	Treatment from private provider	All children for whom symptoms of diarrhoea were reported and who sought treatment	521	-	521	71.9 % (67.2–76.2)
*Acute respiratory infection (ARI)*						
5.	Reported illness	All children	10,006	0.06 %	10,000	7.6 % (6.9–8.4)
6.	Sought treatment	All children for whom symptoms of ARI were reported	761	-	761	78.5 % (74.9–81.8)
7.	Timely treatment	All children for whom symptoms of ARI were reported and who sought treatment	597	0.17 %	596	62.0 % (57.0–66.7)
8.	Treatment from private provider	All children for whom symptoms of ARI were reported and who sought treatment	597	-	597	72.6 % (68.2–76.6)

^a^Weighted sample sizes. Complex survey design was accounted for in calculations of proportions and confidence intervals. 95%CI: 95 % confidence interval

### Multivariable analysis

#### Diarrhoea health-seeking

Adjusted analysis showed that data were consistent with having no association between socio-cultural capital or economic capital and the odds of reporting diarrhoea (Table 3). Female children had lower odds of having had diarrhoea than male children (OR = 0.90), but this association was not significant (*p* = 0.168). The odds of diarrhoea was highest among children 6–11 months old and decreased rapidly for older children, but was not associated with the length of the preceding birth interval. Compared to children of mothers aged 20–24 years, children of teenage mothers (14–19 years old) were more likely to have had diarrhoea. Children living in rural areas were less likely (OR = 0.79, *p* = 0.036) to have had diarrhoea than children from urban areas. Compared to children whose households were interviewed in April, those interviewed in March had twice the odds of reporting diarrhoea (OR = 2.03, *p* < 0.001).

**Table 3:** Adjusted effects of socio-cultural capital and economic capital on child health-seeking behaviours for diarrhoea

Variable		Reported symptoms		Sought treatment		Timely treatment		Private treatment	
	*Sample*	*9,994*		*837*		*517*		*517*	
		*OR (95 % CI)*	*p*	*OR (95 % CI)*	*p*	*OR (95 % CI)*	*p*	*OR (95 % CI)*	*p*
Socio-cultural capital^a^		0.93 (0.80–1.07)	0.305	0.98 (0.71–1.35)	0.890	1.50 (0.98–2.27)	0.059	1.05 (0.70–1.57)	0.802
Economic capital^a^		0.98 (0.81–1.17)	0.787	1.33 (0.96–1.83)	0.082	0.93 (0.60–1.44)	0.747	1.99 (1.26–3.13)	0.003
Child age in months	0–5	0.57 (0.44–0.74)	<0.001	0.83 (0.50–1.39)	0.478	1.04 (0.59–1.83)	0.893	0.88 (0.42–1.81)	0.722
	6–11	1 (ref)		1 (ref)		1 (ref)		1 (ref)	
	12–23	0.51 (0.42–0.63)	<0.001	0.82 (0.54–1.25)	0.351	0.84 (0.49–1.45)	0.527	0.82 (0.45–1.50)	0.578
	24–35	0.30 (0.23–0.38)	<0.001	0.50 (0.31–0.81)	0.005	1.39 (0.71–2.72)	0.334	0.95 (0.45–2.01)	0.893
	36–47	0.14 (0.11–0.19)	<0.001	0.51 (0.28–0.93)	0.028	1.68 (0.77–3.70)	0.194	0.38 (0.17–0.84)	0.018
	48–59	0.12 (0.09–0.17)	<0.001	0.55 (0.29–1.06)	0.074	1.15 (0.42–3.15)	0.785	0.68 (0.28–1.63)	0.383
Child sex	Male	1 (ref)		1 (ref)		1 (ref)		1 (ref)	
	Female	0.90 (0.77–1.04)	0.168	0.97 (0.71–1.34)	0.861	1.11 (0.74–1.67)	0.626	1.50 (0.98–2.30)	0.061
Preceding birth interval	<24 months	1.02 (0.80–1.32)	0825	0.72 (0.44–1.19)	0.200	1.37 (0.71–2.66)	0.349	0.71 (0.35–1.45)	0.348
	24–47 months	1 (ref)		1 (ref)		1 (ref)		1 (ref)	
	≥48 months	0.94 (0.74–1.19)	0.599	1.00 (0.62–1.62)	0.984	1.23 (0.65–2.30)	0.521	1.06 (0.52–2.18)	0.868
	Only child	0.91 (0.72–1.16)	0.455	0.94 (0.60–1.48)	0.790	1.14 (0.58–2.23)	0.704	1.40 (0.66–2.96)	0.377
Mother’s age group	14–19	1.36 (1.00–1.84)	0.043	1.06 (0.63–1.78)	0.826	1.48 (0.70–3.13)	0.299	1.18 (0.53–2.66)	0.686
	20–24	1 (ref)		1 (ref)		1 (ref)		1 (ref)	
	25–29	0.89 (0.72–1.11)	0.302	0.98 (0.65–1.49)	0.941	1.17 (0.70–2.00)	0.556	0.81 (0.44–1.49)	0.498
	30–34	0.93 (0.71–1.22)	0.628	0.76 (0.45–1.28)	0.298	0.90 (0.45–1.80)	0.770	0.97 (0.44–2.13)	0.938
	35–39	0.70 (0.49–1.00)	0.054	1.07 (0.52–2.21)	0.815	0.87 (0.32–2.37)	0.781	0.65 (0.24–1.72)	0.381
	40–49	0.69 (0.40–2.20)	0.192	0.89 (0.26–2.98)	0.873	0.26 (0.07–0.97)	0.045	0.68 (0.16–2.87)	0.601
Mother female head status	Yes	1 (ref)		1 (ref)		1 (ref)		1 (ref)	
	No	0.92 (0.73–1.16)	0.505	0.98 (0.61–1.58)	0.939	0.53 (0.28–0.99)	0.049	1.41 (0.69–2.88)	0.347
Children <5 in household	1	1 (ref)		1 (ref)		1 (ref)		1 (ref)	
	2	0.86 (0.69–1.06)	0.153	0.83 (0.55–1.24)	0.359	0.89 (0.50–1.58)	0.695	0.82 (0.44–1.55)	0.545
	3/4	0.81 (0.62–1.05)	0.108	1.12 (0.61–2.06)	0.720	0.90 (0.42–1.93)	0.786	1.50 (0.63–3.54)	0.356
Region	Urban	1 (ref)		1 (ref)		1 (ref)		1 (ref)	
	Rural	0.79 (0.64–0.99)	0.036	1.29 (0.87–1.90)	0.200	1.74 (1.05–2.87)	0.031	1.15 (0.64–2.06)	0.644
Month of interview	March	2.03 (1.62–2.53)	<0.001					1.06 (0.62–1.80)	0.838
	April	1 (ref)						1 (ref)	
	May/June	1.00 (0.80–1.27)	0.951					0.60 (0.31–1.14)	0.121
Blood in stool	No			1 (ref)		1 (ref)		1 (ref)	
	Yes			2.25 (1.10–4.62)	0.027	0.86 (0.38–1.95)	0.718	0.63 (0.28–1.42)	0.261
Fever in recall period	No			1 (ref)		1 (ref)		1 (ref)	
	Yes			1.60 (1.08–2.38)	0.020	1.33 (0.84–2.11)	0.227	0.87 (0.50–1.51)	0.621
ARI in recall period	No			1 (ref)		1 (ref)		1 (ref)	
	Yes			2.19 (1.37–3.51)	0.001	0.63 (0.38–1.06)	0.082	0.92 (0.53–1.61)	0.773
Timely diarrhoea treatment	No							1 (ref)	
	Yes							2.08 (1.29–3.34)	0.003

*OR* Odds ratio. P-value of Wald test. *ARI* Acute respiratory infection. *95%CI* 95 % confidence interval. Weighted sample sizes reported

^a^Odds of health-seeking behaviour under investigation associated with one unit increase in standardised score

Adjusted analysis of seeking care for diarrhoea, a one-unit increase in economic capital score resulted in an increase of 33 % (*p* = 0.082) in the odds of seeking treatment. There was no association between socio-cultural capital and odds of seeking care for diarrhoea. Children older than 24 months had half the odds of being taken for care than children 6–11 months of age. There was no association between seeking care for diarrhoea and child gender, preceding birth interval, maternal age group, maternal household status and number of children under-five in the household. Children with diarrhoea living in rural areas had higher odds of being taken for care than those living in urban areas, but the association was not significant. Children with blood in the stool, fever and ARI in the recall period had significantly higher odds of being taken for treatment with diarrhoea than children without these additional symptoms.

Two-thirds of children who were taken for treatment with diarrhoea received timely care. A one unit increase in socio-cultural capital was associated with 50 % higher odds of receiving timely care (*p* = 0.059). There was no association between economic capital and the odds of seeking timely diarrhoea care. Child age, gender, preceding birth interval and number of children under-five living in household showed no significant association with the odds of seeking timely care. Children of mothers who were not female heads of household had lower odds of being taken for timely treatment (OR = 0.53, 95%CI: 0.28–0.99). Children from rural areas had 74 % higher odds of being taken for timely care compared to urban children.

Among children for whom diarrhoea care was sought, three quarters were taken to private sector providers. In adjusted analysis, socio-cultural capital was not associated with seeking private treatment. However, a one unit increase in economic capital was associated with a doubling in the odds of private treatment (OR = 1.99, *p* = 0.003). There was some evidence that children in older age groups were less likely to be taken to private providers. Female children had 50 % higher odds of being taken to a private provider compared to male children (*p* = 0.061). Preceding birth interval, mother’s age group, mother’s female head of household status, number of children under-five in the household, region and month of interview were not significantly associated with the odds of approaching private providers. Children for whom timely care was sought had more than twice the odds of receiving private care compared to children for whom care-seeking was delayed (OR = 2.08, *p* = 0.003).

#### ARI health-seeking

Adjusted analyses of the determinants of health-seeking for ARI are presented in Table 4. There was no association between economic capital and odds of reporting ARI. However, a one unit increase in socio-cultural capital was marginally associated with lower odds of reporting ARI (*p* = 0.061). Girls had slightly lower odds of ARI than boys, but this association was not significant. The odds of ARI were highest among 6–11 month old children, and decreased significantly for older children. Children living in rural areas were less likely (OR = 0.73, *p* = 0.018) to have had ARI than children living in urban areas. Compared to children whose households were interviewed in April, children in households interviewed in March had significantly higher odds of ARI than those interviewed in April (OR = 2.62, *p* < 0.001).

**Table 4:** Adjusted effects of socio-cultural capital and economic capital on child health-seeking behaviours for ARI

Variable		Reported symptoms		Sought treatment		Timely treatment		Private treatment	
	Sample	9,999		760		596		596	
		OR (95 % CI)	*p*	OR (95 % CI)	*p*	OR (95 % CI)	*p*	OR (95 % CI)	*p*
Socio-cultural capital^a^		0.85 (0.72–1.01)	0.061	1.20 (0.81–1.77)	0.360	1.23 (0.86–1.78)	0.261	0.95 (0.65–1.39)	0.789
Economic capital^a^		1.12 (0.91–1.37)	0.300	1.06 (0.70–1.61)	0.777	1.18 (0.81–1.73)	0.387	2.66 (1.57–4.52)	<0.001
Child age in months	0–5	0.62 (0.45–0.85)	0.003	1.70 (0.73–3.98)	0.221	1.27 (0.62–2.60)	0.509	0.85 (0.42–1.72)	0.653
	6–11	1 (ref)		1 (ref)		1 (ref)		1 (ref)	
	12–23	0.75 (0.59–0.96)	0.021	0.96 (0.54–1.71)	0.891	1.01 (0.58–1.74)	0.978	0.62 (0.33–1.14)	0.124
	24–35	0.47 (0.36–0.61)	<0.001	0.75 (0.40–1.42)	0.377	1.32 (0.72–2.42)	0.375	0.63 (0.31–1.27)	0.196
	36–47	0.40 (0.31–0.52)	<0.001	0.90 (0.45–1.78)	0.753	0.86 (0.46–1.60)	0.626	0.57 (0.28–1.17)	0.125
	48–59	0.41 (0.31–0.54)	<0.001	0.71 (0.36–1.38)	0.313	1.14 (0.56–1.29)	0.723	0.67 (0.31–1.43)	0.298
Child sex	Male	1 (ref)		1 (ref)		1 (ref)		1 (ref)	
	Female	0.89 (0.76–1.04)	0.142	0.69 (0.46–1.04)	0.076	1.04 (0.72–1.50)	0.854	1.01 (0.69–1.47)	0.964
Preceding birth interval	<24 months	0.87 (0.67–1.12)	0.277	1.41 (0.68–2.94)	0.377	1.28 (0.70–2.34)	0.427	0.66 (0.36–1.23)	0.191
	24–47 months	1 (ref)		1 (ref)		1 (ref)		1 (ref)	
	≥48 months	1.01 (0.80–1.28)	0.944	1.25 (0.72–2.18)	0.402	0.84 (0.50–1.43)	0.519	1.16 (0.65–2.07)	0.623
	Only child	0.74 (0.58–0.94)	0.014	0.88 (0.52–1.48)	0.621	1.16 (0.70–1.91)	0.565	1.25 (0.70–2.23)	0.442
Mother’s age group	14–19	1.22 (0.87–1.71)	0.244	1.06 (0.53–2.14)	0.864	0.85 (0.40–1.82)	0.680	0.57 (0.25–1.34)	0.201
	20–24	1 (ref)		1 (ref)		1 (ref)		1 (ref)	
	25–29	1.05 (0.84–1.33)	0.655	0.88 (0.51–1.50)	0.637	1.41 (0.84–2.36)	0.193	0.92 (0.51–1.67)	0.787
	30–34	0.97 (0.74–1.27)	0.842	1.33 (0.70–2.56)	0.380	1.10 (0.60–2.03)	0.747	0.56 (0.29–1.06)	0.076
	35–39	0.79 (0.56–1.12)	0.195	1.18 (0.53–2.64)	0.687	1.89 (0.85–4.19)	0.118	0.90 (0.35–2.32)	0.826
	40–49	0.74 (0.43–1.26)	0.270	0.84 (0.27–2.59)	0.761	1.24 (0.35–4.41)	0.735	0.40 (0.11–1.49)	0.171
Mother female head status	Yes	1 (ref)		1 (ref)		1 (ref)		1 (ref)	
	No	0.95 (0.74–1.23)	0.725	1.18 (0.63–2.13)	0.578	1.40 (0.80–2.46)	0.235	1.23 (0.64–2.35)	0.531
Children <5 in household	1	1 (ref)		1 (ref)		1 (ref)		1 (ref)	
	2	0.94 (0.76–1.16)	0.554	1.37 (0.85–2.20)	0.190	0.85 (0.54–1.35)	0.485	0.96 (0.59–1.55)	0.866
	3/4	0.88 (0.64–1.18)	0.386	1.09 (0.53–2.24)	0.822	0.63 (0.33–1.21)	0.163	1.79 (0.80–4.01)	0.158
Region	Urban	1 (ref)		1 (ref)		1 (ref)		1 (ref)	
	Rural	0.73 (0.56–0.95)	0.018	0.81 (0.49–1.34)	0.406	1.56 (0.98–2.50)	0.061	1.96 (1.18–3.27)	0.010
Month of interview	March	2.62 (2.09–3.29)	<0.001					0.96 (0.60–1.54)	0.871
	April	1 (ref)						1 (ref)	
	May/June	0.62 (0.46–0.85)	0.003					0.52 (0.26–1.07)	0.075
Fever in recall period	No			1 (ref)		1 (ref)		1 (ref)	
	Yes			3.24 (2.06–5.10)	<0.001	1.29 (0.79–2.10)	0.300	0.90 (0.52–1.56)	0.702
Diarrhoea in recall period	No			1 (ref)		1 (ref)		1 (ref)	
	Yes			0.79 (0.50–1.25)	0.312	0.85 (0.54–1.34)	0.485	0.71 (0.46–1.10)	0.122
Timely ARI treatment	No							1 (ref)	
	Yes							1.15 (0.75–1.76)	0.532

*OR* Odds ratio. P-value of Wald test. *ARI* Acute respiratory infection. *95%CI* 95 % confidence interval. Weighted sample sizes reported

^a^Odds of health-seeking behaviour under investigation associated with one unit increase in standardised score

Adjusted analysis of seeking care for ARI showed that a one unit increase in socio-cultural capital score was associated with a 20 % increase in the odds of seeking treatment, but this association was not significant (*p* = 0.360). There was no association between economic capital and odds of seeking care for ARI. Child age was not significantly associated with seeking ARI treatment. Girls had 31 % lower odds of being taken for treatment compared to boys (*p* = 0.076). Children with fever in the recall period had more than triple the odds of being taken for treatment with ARI compared to children without fever (*p* < 0.001).

Two-thirds of children who were taken for ARI treatment received timely care. Neither socio-cultural nor economic capital was significantly associated with the odds of seeking timely treatment for ARI. However, the direction of the association was positive for both variables. Child age, gender, preceding birth interval, maternal age group and number of children under-five living in household showed no significant association with the odds of seeking timely care. Children from rural areas had 56 % higher odds of being taken for timely care compared to urban children (*p* = 0.061).

Among children for whom ARI care was sought, three-quarters were taken to private-sector providers. In adjusted analysis, socio-cultural capital was not associated with seeking private treatment. However, a one unit increase in economic capital was associated with more than twice the odds of private treatment (OR = 2.66, *p* < 0.001). Compared to children living in urban areas, children from rural areas had double the odds of being taken for private ARI care (OR = 1.96, *p* = 0.010). Receiving private care for ARI was not associated with timeliness of seeking ARI care.

### Mediation analysis

#### Diarrhoea health-seeking

The results of mediation analysis with the adjusted models for health-seeking in response to diarrhoea are presented in Table 5. Neither socio-cultural capital nor economic capital was significantly associated with seeking treatment for diarrhoea; the total effect of socio-cultural capital on this outcome was also not significant and mediation analysis was not applicable. Among children for whom care was sought, a one unit increase in socio-cultural capital resulted in a 7 percentage point increase in the probability of seeking timely treatment (95 % CI: 1–14). The total effect of socio-cultural capital was entirely direct (0 % was mediated by economic capital). Socio-cultural capital was not associated with the odds of seeking private care, but there was a strong association between economic capital and this outcome. The total effect of socio-cultural capital on the probability of seeking private care for positive; a one unit increase in socio-cultural capital resulted on average in a 5 percentage point increase in the probability of seeking private care among children for whom care was sought. The vast majority of this total effect (92 %) was mediated by economic capital.

**Table 5:** Mediation: adjusted effects of socio-cultural capital and economic capital on child health-seeking behaviours

Outcome	(1) Direct effect of socio-cultural capital	(2) Direct effect of economic capital	(3) Total effect of socio-cultural capital	(4) % of total effect of socio-cultural capital mediated by economic capital
	*OR (95 % CI)*	*OR (95 % CI)*	*ΣΔp (95 % CI)*	*% (95 % CI)*
*Diarrhoea*				
Sought treatment	0.98 (0.71 to 1.35)	1.33 (0.96 to 1.83)	0.02 (−0.03 to 0.08)	Not applicable
Timely treatment	1.50 (0.98 to 2.27)	0.93 (0.60 to 1.44)	0.07 (0.01 to 0.14)	0 %
Private treatment	1.05 (0.70 to 1.57)	1.99 (1.26 to 3.13)	0.05 (0.00 to 0.10)	92 % (0 % to 100 %)
*ARI*				
Sought treatment	1.20 (0.81 to 1.77)	1.06 (0.70 to 1.61)	0.03 (−0.02 to 0.07)	Not applicable
Timely treatment	1.23 (0.86 to 1.78)	1.18 (0.81 to 1.73)	0.07 (0.00 to 0.13)	28 % (15 % to 100 %)
Private treatment	0.95 (0.65 to 1.39)	2.66 (1.57 to 4.52)	0.06 (0.01 to 0.11)	99 % (64 % to 100 %)

*OR* Odds ratio associated with one unit increase in score. *ARI* Acute respiratory infection. *95%CI* 95 % confidence interval. *ΣΔp* Total effect of socio-cultural capital expressed as sum of the changes in probability of outcome based on both indirect (mediated by economic capital) and direct effects

#### ARI health-seeking

Table 5 shows that neither socio-cultural capital nor economic capital was significantly associated with seeking treatment for ARI. The total effect of socio-cultural capital on this outcome was also not significant and mediation analysis was not applicable. Among children for whom ARI care was sought, the total effect of a one unit increase in socio-cultural capital was a 7 percentage point increase in the probability of seeking timely treatment (95%CI: 0–13). On average, 28 % of this effect was mediated by economic capital. Socio-cultural capital was not associated with the odds of seeking private care, but there was a strong association between economic capital and this outcome. The total effect of socio-cultural capital on the probability of seeking private care was positive; a one unit increase in socio-cultural capital resulted, on average, in a 6 percentage point increase in the probability of seeking private care among children for whom ARI care was sought. Economic capital was the primary mechanism of this total effect.

## Discussion

Based on the systematic review, this is the first analysis to examine curative health-seeking for child illness in a nationally-representative sample from Egypt using multivariable analysis, and also the first in-depth analysis of these outcomes based on the 2008 DHS. The prevalence of both illnesses among children under-five years in the two-week recall period was approximately 8 %. However, more than half of the children who had either diarrhoea or ARI suffered from two or more illness symptoms (diarrhoea, ARI and/or fever). Reporting of diarrhoea or ARI was not independently associated with either measure of SEP. Younger children, those living in urban areas (potentially related to informal and slum housing) and interviewed earlier in the year were more likely to have had diarrhoea and ARI. However, it is not possible to determine whether this difference in period prevalence is a result of different incidence, illness duration, illness severity, or an artefact of reporting related to perception of illness.

Care was sought for three-fifths of children with diarrhoea and four fifths of children with ARI. In case of both illnesses, neither of the two latent SEP variables was strongly associated with seeking care and the total effect of socio-cultural capital was not significant. For both diarrhoea and ARI, two-thirds of children for whom care was sought obtained care within a day of the onset of illness. Private sector providers were approached for the majority of care for both illnesses. Mediation analysis showed that for both illnesses, socio-cultural capital had a significant positive total effect on seeking timely care and that this effect was largely, if not completely, the result of its direct effect. On the other hand, the significant positive total effect of socio-cultural capital on the choice of private provider for both illnesses was almost entirely mediated by economic capital.

This study showed that care-seeking for child illnesses in Egypt was not strongly socio-economically patterned. However, among children who were taken for care, those with higher parental socio-cultural capital scores were more likely to receive timely care and those with higher household economic capital were more likely to be taken to private providers. These findings are difficult to directly compare with studies from other countries due to differences in study methodologies and the importance of context, particularly health system and financing, to shaping any observed gradients. The main objective of this study was to differentiate the effects of socio-cultural capital and economic capital as determinants of inequalities in the various steps of health-seeking in Egypt.

Several results of this study are notable and help interpret the findings. Younger children and children with more than one illness during the recall period had higher odds of seeking care. These factors may be related to perceived vulnerability and/or severity of illness, which in turn necessitated seeking medical care. Support for this explanation is bolstered by findings that children with either illness who had fever during the recall period were marginally more likely to seek timely treatment.

While the effect was not significant, the presence of multiple illnesses was negatively associated with the odds of seeking private care. For both illnesses, children for whom timely care was not sought were less likely to be taken to private providers. This effect may be a result of waiting for spontaneous resolution among families that do not have the resources to access private care. The preference for public care when such improvement was not seen may be either due to perceptions of better clinical capacity in the public sector, or as a more affordable alternative. Public providers were more likely to be approached by families of children with more severe illness, regardless of care-seeking timeliness. If true, this effect could have arisen due to various characteristics of the public compared to the private sector, such as clinical capacity, availability/affordability of medication, suitability of opening hours (particularly in emergencies), capacity for referral to higher levels of care, and/or perception of better quality of care.

In this analysis, neither measure of SEP was directly constructed from the child’s perspective. Sex is one inherent characteristic of the child which may act as an indicator of the child’s societal status. A study of child curative health-seeking in Minia found that ill girls were less likely to be taken to a medical provider than boys [30]. Likewise, crude tabulations in the 2008 DHS report showed that compared to girls, slightly higher proportions of boys were taken for treatment (ARI: 77 % and 68 %; diarrhoea: 57 % and 54 %, respectively) [1]. Our study did not identify any significant associations between child sex and illness reporting or help-seeking in multivariable analyses and found that compared to boys, private diarrhoea care was marginally more likely to be sought for girls.

### Limitations

The survey response rate was high and extent of missing data was minimal. However, this analysis had several limitations. Socio-cultural capital is a measure of parental knowledge, awareness and collaboration, whereas economic capital captures the household-level availability of financial resources and expenditure preferences. These two latent variables were created for female respondents of reproductive age residing in sampled households [17], and therefore were only available for children whose mothers were in this group and were alive. There are no reliable recent estimates of the proportion of children whose mother is not alive, but it is likely that such children are more vulnerable to illness and may also have more limited access to health services.

The construction of outcomes in this study was dependent on women’s self-report of child illness and health-seeking actions. The perception and accurate recall of illness among young children has numerous issues of reliability and validity. Accurate reporting of symptoms depends largely on the respondent’s knowledge of these symptoms, and on the subjective perception of illness by the respondent and/or other household members. The two-week recall period for recall of ARI as a proxy for pneumonia was found to have acceptable specificity for the purpose of monitoring trends in the proportion of children who receive medical care in Egypt [31]. Mothers have been shown to correctly recognise rapid or difficult breathing among their children [32].

No recent studies have assessed the reliability and validity of reports of timeliness of care and provider type in Egypt. To reduce the potential outcome misclassification, this outcome was dichotomised, considering health-seeking within one day of illness onset as timely. However, we do not wish to imply that seeking care beyond one day after illness onset meant that care-seeking was suboptimal. It could have reflected a situation when initial symptoms were mild and self-limiting, but which progressively became serious enough to seek treatment. A second issue with the assessment of timeliness is that in cases when the illness onset occurred on the day before the survey or on the day of survey, sufficient time had not yet elapsed for this outcome to occur. This limitation extends to the first dimension (seeking any care) in that this outcome may still occur in the future, and is therefore misclassified as not having sought care. While the questionnaire asked whether the child was still ill at the time of the survey, information about the duration of the illness episode at the time of survey was not collected. Only the availability of both these items of information would allow for a more precise analysis of seeking any care and seeking timely care among the group of children who had the illness long enough to have sought care, and for a comparison of illness duration between children who were taken for treatment and those who were not.

The reason this study of health-seeking for children’s curative care began with an examination of the SEP gradients in reporting illness is due to the possibility of socio-economic patterning in perception of childhood illness. Despite ill-health concentrating among the poorest and most vulnerable groups in any society, a phenomenon of illness reporting and use of healthcare among richer groups is generally observed [33]. This phenomenon has been partly attributed to a lower level of sensitivity to illness among the vulnerable groups most affected by ill-health. It has been documented in Egypt [34], and posited as one of the mechanisms underlying the socio-economic gradients in infant and child mortality [35]. The analysis of factors associated with health-seeking behaviours in this study did not allow for a direct assessment of socio-economic patterns in illness perception and reporting which could underlie this important bias. It is possible that the prevalence of illness was under-perceived and/or under-reported for children living in households with lower SEP. The crude tabulations of diarrhoea and ARI period prevalence in the EDHS report showed that children living in poorer households were slightly more likely to have been ill [1]. However, the multivariable analysis presented here failed to identify a strong independent effect of either SEP latent variable on illness reporting.

The correct estimation of associations in the presented models relies on the absence of unmeasured confounding. Perceived illness severity might be a source of residual confounding. The data contain no objective or subjective indicators of whether severity illness in children necessitated medical attention. As a proxy, we used the reported occurrence of other illnesses during the recall period in multivariable analysis. While the survey provided no information about whether such multiple illnesses occurred as a part of the same episode or captured the chronological sequence of their onset, a study among Indian and Nepali children showed that diarrhoea and acute lower respiratory illness occurred simultaneously more than by chance alone [28]. The results showed that presence of other illnesses was a strong determinant of health-seeking behaviours, and that while imperfect, such period co-morbidities appeared to capture an element of child ‘frailty’ during the recall period [27, 36].

Lastly, the data used in this analysis were collected before the socio-political situation in Egypt underwent dramatic changes starting in early 2011. While these developments may have influenced the patterns of child illness prevalence, as well as supply of and demand for curative care, the results of our analysis can be used in the future in conjunction with updated data to explicate the effect of these events on the coverage and socio-economic determinants of health-seeking for ill children.

## Conclusion

Two research directions could be pursued to improve the understanding of whether and how health-seeking behaviours contribute to the observed socio-economically patterned inequalities in child health outcomes. First, children in rural areas were more likely than urban dwellers to obtain timely care, and care was more likely to be sought from private providers. It would be important to determine whether, compared to urban regions, the patterns of health-seeking in rural areas are due to different perceptions of symptoms, higher severity of illness, larger variation in the perceived quality of care between public and private providers, or lower availability of public care in rural regions. Second, a better understanding of the quality of care children actually receive when they seek medical care is needed. In the scope of this study, seeking care does not mean that adequate diagnosis and treatment was received; and for diarrhoeal illness, not seeking medical care does not mean that appropriate treatment (e.g., in the form of home-made oral rehydration solution) was not given. There is some evidence that male children were more likely to seek and receive appropriate diarrhoea treatment from public providers [37], and such gradients in quality of care may exist based on other socio-economic characteristics within the sample of children for whom care is sought. Therefore, provision of quality of care in a very broad sense of the concept should be explored, including the level of communication between the care takers and the health professionals, dignity and respect, laboratory and medications tests prescribed and purchased, and adherence to treatment instructions.
